# An alternative mebendazole formulation for cystic echinococcosis: the treatment efficacy, pharmacokinetics and safety in mice

**DOI:** 10.1186/s13071-014-0589-0

**Published:** 2014-12-10

**Authors:** Cong-Shan Liu, Hao-Bing Zhang, Wen Lei, Chao-Wei Zhang, Bin Jiang, Qi Zheng, Jian-Hai Yin, Xiu-Min Han

**Affiliations:** National Institute of Parasitic Diseases, Chinese Center for Disease Control and Prevention, Key Laboratory of Parasite and Vector Biology, MOH, WHO Collaborating Centre for Malaria, Schistosomiasis and Filariasis, Shanghai, 200025 China; Qinghai Institute for Endemic Disease Prevention and Control, Xining, 811602 Qinghai China

**Keywords:** Mebendazole oily suspension, Cystic echinococcosis, Treatment efficacy, Pharmacokinetics, Histopathology examination, Liver function test

## Abstract

**Background:**

Cystic echinococcosis is a serious zoonotic infection worldwide caused by metacestodes of *Echinococcus gruanulosus*. Mebendazole and albendazole are the only two drugs used in the treatment of this disease with cure rates only about 30% due to the poor oral absorption. Thus an alternative treatment for this disease is needed.

**Methods:**

A mebendazole oily suspension (MBZ-OS) was prepared and orally administrated to mice infected with echinococcus cysts for 8 months at 12.5 mg/kg and 25 mg/kg for 14 consecutive days. Mebendazole suspended in 1% tragacanth (MBZ-1% tragacanth) served as treated control. In addition, liver and serum samples were collected from these treated mice (25 mg/kg) for histopathology examination and liver function test. For pharmacokinetic analysis, plasma, parasite (cyst wall and cyst fluid) and tissue samples were collected at 0.25, 0.5, 1, 2, 4, 8, 16 and 24 h after orally administrating MBZ-OS and MBZ-1% tragacanth to *E. granulosus*-infected mice at 25 mg/kg. These samples were then processed and quantitatively analyzed by HPLC.

**Results:**

The administration of MBZ-OS resulted in a treatment efficacy with the cyst weight reductions higher than 80%, significantly better than the corresponding MBZ-1% tragacanth groups. The better treatment efficacy of MBZ-OS was related to the higher drug concentration in plasma, parasites and tissues. It was also shown that the injury of the liver was not significantly altered by taking MBZ-OS compared to the untreated control.

**Conclusion:**

These findings demonstrate that MBZ-OS is a promising new formulation of MBZ for treatment of hydatid diseases without showing significantly liver toxicity.

**Electronic supplementary material:**

The online version of this article (doi:10.1186/s13071-014-0589-0) contains supplementary material, which is available to authorized users.

## Background

Cystic echinococcosis (CE) is a life-threatening disease with serious public health and economic concern worldwide. Infections with *Echinococcus granulosus*, the causative agent of CE, occur globally and mostly in the Mediterranean area, South America, Russia, western China, Europe and Japan [1,2]. In China, with approximately 380,000 cases nationwide, CE is endemic in at least 23 provinces [3-5] and at least 50 million individuals are under the risk of this disease [6]. The proliferation of metacestodes mainly located in liver and lung, leading to the space-occupying lesions. However, the initial phase of the infection is always asymptomatic for many years or even permanently. Without effective treatment, the development of cysts will eventually result in organ malfunction and even death in many cases [7-9]. The preferred treatment strategies for CE are surgical resection of the parasite mass and puncture, aspiration, injection, re-aspiration (PAIR). The other option is chemotherapy with albendazole (ABZ) or mebendazole (MBZ), the only two drugs recommended by WHO. Both surgery and PAIR are usually combined with these two benzimidazoles, while chemotherapy is the only option in inoperable and recrudescent cases [9]. Moreover, for the great majority of patients in the resource-poor remote areas in China, anti-parasitic treatment remains the primary choice [10].

ABZ and MBZ are considered to have parasitocidal effect *in vitro* [11,12]. Nevertheless, according to the reports from the clinical trials, the cure rates of MBZ and ABZ in the treatment of CE were about 30% [3,7,8,13-15]. The unsatisfied therapeutic results are commonly attributed to the poor drug absorption rate which results in low drug concentartions in plasma and hydatid cysts after oral administration [16]. In addition, for MBZ, the inactive metabolites produced in the liver could be another possible reason contributing to the low active drug level [17]. In order to improve cure rate, these two benzimidazoles were recommended to be taken together with a fat-containing meal [9]. In some reports, suspending benzimidazoles in oils increased the solubility [18], the drug concentration in plasma, the bioavailability [18-22] and even the treatment efficacy for infected animals [18,22,23]. In China, the albendazole-emulsion was proved effective [24,25] and has already been widely used in clinic. Due to the better effect of MBZ against *E. granulosus* metacestodes *in vitro* and in experimental therapy proceeded in mice than ABZ [11,12,26,27], a superior improvement of MBZ treatment efficacy could be achieved by increasing the bioavailability.

Until now, some progress has been achieved on the novel formulations of MBZ. Solid dispersion with polyethylene glycol [28,29], β-cyclodextrin compounds (β-CD) [30], capsule [31] or drug-povidone complexes [16] were tested in rodents and showed enhanced bioavailabilities compared with the parent drugs. And the improving efficacy of solid dispersion with polyethylene glycol and drug-povidone complexes for CE was also confirmed in some studies [32,33]. However, these researches for MBZ formulations have not been continued and eventually translated into clinical applications. Based on our previous study [18], we prepared a MBZ oily suspension with oleic acid, glycerol trioleate, soy bean oil, some surfactants and preservatives in the present study. Subsequently, the treatment efficacy, the drug concentrations in plasma, parasite and tissues, and the influence on the liver were evaluated.

## Methods

### Chemicals

MBZ powder with the purity over 99.0% was purchased from Hanjiang Pharm. Co. (Hanzhong, China). MBZ standard and ABZ served as internal standard were purchased from Sigma-Aldrich Co. (St. Louis, UAS). The glycerol trioleate, oleic acid and soybean oil were provided by Qianwei Oil Technology Co., Ltd. (Shanghai, China). All other chemicals were of the analytical pure grade.

### Parasites, animals and infection

Liver hydatid cysts were obtained from newly slaughtered sheep at abattoirs in Xining, Qinghai province, China. Protoscoleces of *E. granulosus* were collected aseptically from the cysts, and kept in cyst fluid at 4°C for not more than 3–4 days before use. Prior to infection, the collected protoscoleces were rinsed 5–8 times with Hanks’ balanced salt solution (HBSS) containing penicillin (500 U/ml) and streptomycin (500 U/ml). Viability of the PSCs was confirmed by visual inspection through inverted microscopy after staining by 0.1% methylene blue. A protoscoleces survival rate of more than 95% was necessary before the infection. Kunming strain mice were purchased from SLAC Laboratory Animal (Shanghai, China). Animals were kept at the animal facility and had free access to rodent food and tap water throughout the study. After one week acclimatization, the female mice with body weight of 18–22 g were inoculated intraperitoneally with 2,000 protoscoleces individually.

### Preparation of MBZ oily formulation

The MBZ oily formulation (MBZ-OS) were prepared by suspending MBZ in the mixture of oleic acid, glycerol trioleate, soybean oil, span 80, tween 80 and sorbic acid at a concentration of 2.5 and 1.25 g/l (w/v) respectively, while MBZ in 1% tragacanth (MBZ-1% tragacanth) at the same concentration served as treated control. The volume of each drug preparation administered to mice was 10 ml/kg. All the drug suspensions were made with bowl mill (Nanjing, China) at 220 rpm for 1 h.

### Effect against secondary cysts of *E. granulosus*

Seventy-one mice inoculated with *E. granulosus* protoscoleces for 8 months were divided into 5 groups of 10–17 mice. Two groups were treated orally with MBZ-OS at a daily dose of 25 mg/kg and 12.5 mg/kg for 14 consecutive days respectively, and two groups were treated with MBZ-1% tragacanth as the same scheme. The remaining one group of 17 mice infected but untreated served as control. Mice were sacrificed two weeks post-treatment. Then the cysts in the peritoneal cavity were isolated and removed for weighing. The efficacy was assessed by mean cyst weight and mean cyst weight reduction as previously described [18].

### Drug concentrations and pharmacokinetics of MBZ formulation

#### Collection of plasma, cyst wall, cyst fluid and tissues samples

Forty-eight mice inoculated with *E. granulosus* protoscoleces for 8 months were divided into two groups, one was administered orally with MBZ-OS at a single dose of 25 mg/kg, and the other was treated orally with the same dose of MBZ-1% trangacanth. Subgroups of 3 mice in each group were bled at 0.25, 0.5, 1, 2, 4, 8, 16 and 24 h post-administration. The plasma samples were collected after centrifugation at 2583 x g for 15 minutes and then stored immediately at −20°C pending HPLC analysis.

Then the hydatid cyst (parasite), heart, liver, spleen, lung, kidney, intestine and brain of each mouse were rapidly excised after blood collection at the same time intervals, while the cyst fluid was drawn from the hydatid cyst by syringe, and then stored at −20°C. The tissues were immediately washed twice with normal saline, wiped with filter paper, weighed and homogenized with 1.0 ml of normal saline. The tissue samples were centrifuged at 2583 x g for 15 minutes. Then the supernatant was collected and then stored at −20°C.

#### Quantitative analysis by HPLC

For MBZ assay, 0.5 ml plasma, cyst wall, cyst fluid and mice’s tissue samples with ABZ as internal standard was extracted with Oasis HLB Cartridges (Waters, USA). The final collection of methanol elution was dried and redissolved in methanol. All samples were filtrated with 0.45 μm membrane filter before injection.

The system of instrument consisted of a 1525 Binary HPLC Pumps, a 717 plus auto sampler and a 2457 Dual λ Absorbance Detector (Waters, USA). The assay of MBZ was performed in a 5 μm C_18_ 250 × 4.6 mm column (Beckman Coulter, USA) and a mobile phase containing 350 ml of 0.05 M ammonium sulfate and 650 ml of methanol at a flow rate of 1 ml/min. The MBZ concentration was measured by its absorbance at a wavelength of 289 nm. The details and validation of the analytical methodology can be seen in Additional file 1.

#### Pharmacokinetic parameters

Using the non-compartmental model present in DAS 2.0 (Drug Analyze System, Shanghai University of T.C.M, China), the pharmacokinetic parameters of AUC_0-t_ (the area under the drug concentration-time curve), C_max_ (the peak concentration of the drug) and T_max_ (time to the drug peak concentration) were estimated. The value of relative bioavailability (F) was calculated by the following formula:

$$ F= AU{C}_{0-t}\kern0.5em  of\kern0.5em MBZ- OS/ AU{C}_{0-t}\kern0.5em  of\kern0.5em MBZ-1\%\  trangacanth $$

### Influence of MBZ-OS on the liver of *E. granulosus*-infected mice

#### Liver function test

Before the mice were sacrificed for evaluating the treatment effect of MBZ-OS against secondary cysts of *E. granulosus*, the blood sera of 10 mice respectively from MBZ-OS and MBZ-1% trangacanth at a dose of 25 mg/kg, and untreated control group were collected and immediately ready for the liver function test. In order to observe the liver injury of mice infected with *E. granulosus* cysts, the blood of normal mice, which were kept for the same 8 months was also collected and served as uninfected group. The liver function test was carried out by Adicon Clinical Laboratories (Shanghai, China) with Automatic Chemistry Analyzer 640 (OLYMPUS, Japan). The total protein (TP), albumin (ALB), globulin (GLB), total bilirubin (TBIL), direct bilirubin (DBIL), indirect bilirubin (IBIL), alkaline phosphatase (ALP), alanine transaminase (ALT), aspartate transaminase (AST) were analyzed with Automatic Chemistry Analyzer 640 (Olympus, Japan).

#### Histopathology examination

The livers of 5 mice from the same two treated groups, untreated control group and uninfected group corresponding to liver function tests were rapidly excised, washed twice with normal saline and fixed in 10% neutral buffered formal. The fixed samples were dehydrated in ascending series of ethanol, embedded in paraffin, cut into 5 μm sections, and stained with hematoxylin and eosin (H + E) for microscopic examination. For statistical purposes, the severity of histopathological changes was measured on a semi-quantitative scale scored in four categories according to the intensity of alterations: without alteration (0), slightly altered (1), intermediately altered (2) and strongly altered (3).

### Statistical analysis

The difference of mean cyst weight and liver function test results were analyzed by ANOVA in SPSS 17.0 and *P* < 0.05 was considered statistically significant.

### Ethics approval

Animal care and all animal procedures were carried out in compliance with the Guidelines for the Care and Use of Laboratory Animals produced by the Shanghai Veterinary Research Institute. The study was approved by the Ethics Committee of the Institute of Parasitic Diseases, Chinese Center for Disease Control and Prevention. The license number was IPD-2012-2.

## Results

### Effect against secondary cysts of *E. granulosus*

In the four groups orally treated with MBZ-OS and MBZ-1% tragacanth at a daily dose of 12.5 mg/kg and 25 mg/kg for 14 days, the mean cyst weights were all lower than that of the untreated control. For MBZ-1% tragacanth groups at 25 mg/kg and 12.5 mg/kg, the cyst weight reductions were 48.9% and 2.2% respectively (Table 1). And the difference between MBZ-1% tragacanth groups and untreated control groups was not statistically significant (*P* > 0.05). However, the mean cyst weights of MBZ-OS groups were significantly lower than that of the untreated control group (*P* < 0.05) with mean cyst weight reductions of 90.2% at 25 mg/kg and 82.6% at 12.5 mg/kg (Table 1). During the experiment, we had some inevitable loss due to the long- term administration. One mouse died in the MBZ-1% tragacanth group at 12.5 mg/kg and one in the MBZ-OS group at 25 mg/kg. But this did not affect the calculation of mean cyst weight.

**Table 1:** **Mean ± SD (g) and reduction (%) of the hydatid cysts weigh recovered from infected mice from the untreated control and treated groups MBZ-1% and MBZ-OS both dosed at 12.5 and 25 mg/kg daily during 14 days**

**Groups**	**Dose (mg/kg per day × 14 days)**	**No. of mice**	**Mean cyst weight g (SD)**	**Reduction of cyst weight (%)**
Untreated control	−	17	0.92 (1.22)	
MBZ-1% tragacanth	25	16	0.47(0.69)	48.9
	12.5	16	0.90 (1.15)	2.2
MBZ-OS	25	10	0.09 (0.07)*	90.2
	12.5	12	0.16 (0.13)*	82.6

*P < 0.05 vs. untreated control.

### MBZ concentration in plasma, cyst fluid, cyst wall and tissues of mice

**Figure 1:**
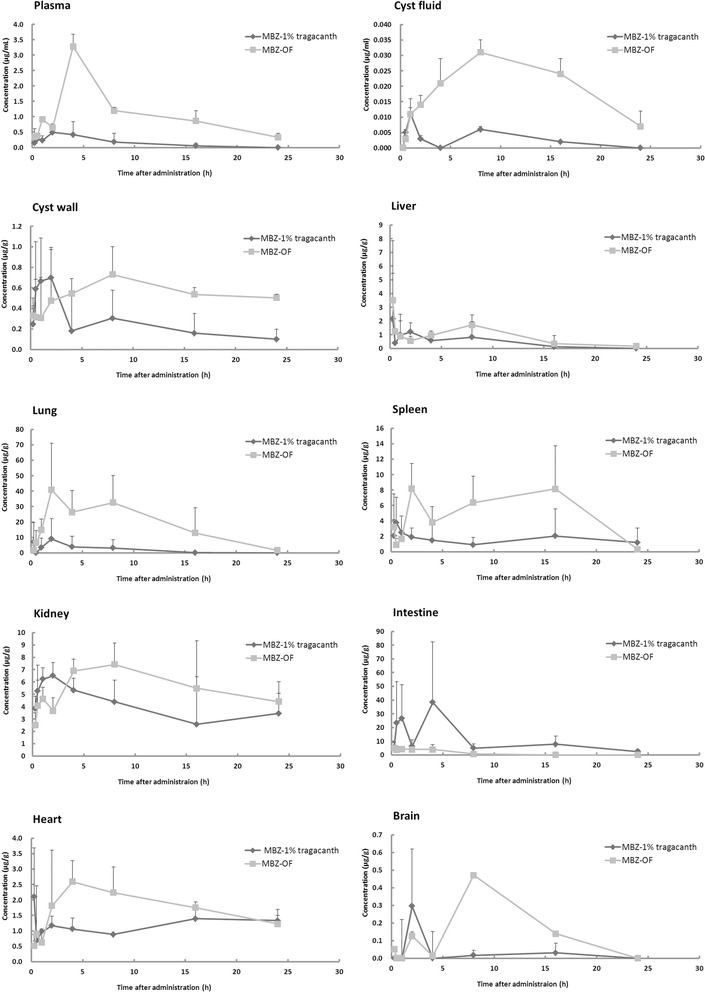
**Mebendazole (MBZ) concentration in plasma and tissues of** ***E. granulosus*** **infected mice orally administrated with MBZ-OS and MBZ-1% tragacanth at a single dose of 25 mg/kg.**

It can be seen from Figure 1 that, both of the MBZ-OS and MBZ-1% tragacanth were absorbed by the parasite and tissues of the mice. After orally administrating MBZ-1% tragacanth to mice at a single dose of 25 mg/kg, a concentration peak of 0.27 ± 0.15 μg/ml showed at 0.5 h post-treatment in the plasma, then the highest MBZ concentration of 0.49 ± 0.23 μg/ml at 2 h (Figure 1). Thereafter, the MBZ concentration declined significantly and maintained at low level up to 24 h. On the other hand, a concentration peak of 0.92 ± 0.01 μg/ml and highest MBZ concentration of 3.28 ± 0.40 μg/ml were emerged in plasma after oral administration of MBZ-OS. Obviously, the MBZ concentration in MBZ-OS group was higher than MBZ-1% tragacanth. And in MBZ-OS group, the time reaching to the plasma peaks was somewhat delayed compared to MBZ-1% tragacanth group.

Similar to that of the plasma, the peak concentration of MBZ-1% tragacanth was 2.11 ± 1.58 μg/g at 0.25 h and then maintained about 1 μg/g in hearts. The highest concentration (2.59 ± 0.68 μg/g) of MBZ-OS emerged at 4 h, and then slowly declined to about 1 μg/g (Figure 1).

As seen in Figure 1, the concentration-time profiles of cyst fluid and cyst wall were almost same. In detail, the first concentration peaks of 0.011 ± 0.002 μg/ml and 0.70 ± 0.3 μg/g had been detected in cyst fluid and cyst wall after 1–2 h post-administration after administrating MBZ-1% tragacanth, and then the second peaks at 8 h (0.006 ± 0.001 μg/ml and 0.30 ± 0.27 μg/g, respectively). In contrast, the MBZ concentration of MBZ-OS increased with time and the highest concentration peaks of 0.031 ± 0.004 μg/ml and 0.73 ± 0.27 μg/g emerged at 8 h in cyst fluid and cyst wall. Thereafter, the drug levels declined but still higher than MBZ-1% tragacanth till the end of the experiment period.

In the liver, the highest MBZ concentrations of MBZ-OS and MBZ-1% tragacanth were 2.16 ± 3.32 μg/g and 3.25 ± 4.33 μg/g at 0.25 h. Then the second concentration peaks of 1.12 ± 0.56 μg/g and 1.71 ± 0.74 μg/g were found at 2 h and 8 h post-administration. According to the concentration-time profile of the lung, the highest drug concentration of MBZ-1% tragacanth was 9.05 ± 13.21 μg/g, and 40.8 ± 30.28 μg/g at 2 h for MBZ-OS. During the 24 h post-administration, the drug concentrations of MBZ-OS were higher than that of MBZ-1% tragacanth in spleen and kidney, with a delayed time reaching the drug peak concentration (Figure 1). The peak concentration of MBZ-1% tragacanth was 0.30 ± 0.32 μg/g at 2 h and then decreased in the brain. For MBZ-OS, there were two concentration peaks (0.13 ± 0.02 μg/g at 2 h and the second 0.47 ± 0.03 μg/g at 8 h).

In contrast to the plasma, parasite and other tissues, the drug concentration of MBZ-1% tragacanth (38.3 ± 44.01 μg/g) in intestine was higher than that of MBZ-OS (5.04 ± 3.68 μg/g). And at the end of 24 h, the drug concentration of MBZ-OS group declined to 0.01 ± 0.00 μg/g while that of MBZ-1% tragacanth was still as high as 2.47 ± 1.38 μg/g.

### Pharmacokinetic parameters of plasma, cyst fluid, cyst wall and tissues of mice

According to the MBZ concentration-time curve obtained from *E. granulosus*-infected mice orally administrated with MBZ formulations at a single dose of 25 mg/kg, T_max_, C_max_, AUC_0-t_ and F were calculated and listed in Table 2. In plasma, T_max_ values of MBZ-OS and MBZ-1% tragacanth were 2.6 ± 1.1 μg/ml and 3.3 ± 1.1 μg/ml, respectively. But for parasite and tissues, the mean T_max_ values were numerically different between two treated groups. It was found that the C_max_ values in the MBZ-OS group were 0.3-4.9 times higher than the MBZ-1% tragacanth group in plasma, cyst fluid, liver, lung, spleen, brain, nearly the same in kidney and 0.6-4.6 times lower in cyst wall and intestine. In addition, the AUC values were 1.5-9.3 times of the MBZ-1% tragacanth group in plasma, parasite and most tissues but not intestine.

**Table 2:** **Pharmacokinetic parameters of MBZ-OS and MBZ-1% tragacanth in plasma and tissues of mice after oral administration at a single dose of 25 mg/kg**

**Plasma, Parasite and Tissues**		**Mean value (SD) of pharmacokinetic parameters (MBZ-1% tragacanth/MBZ-OS)**						
		**T**_**max**_^**a**^ **(h)**		**C**_**max**_^**b**^ **(μg/ml or μg/g)**		**AUC**_**0-t**_^**c**^ **(μg/ml × h or μg/g × h)**		**F** ^**d**^
Plasma		2.6 (1.1)	3.3 (1.1)	0.7 (0.2)	4.1 (1.7)	3.8 (1.9)	27.1 (0.9)	7.1
Parasite	Cyst wall	0.8 (0.3)	6.7 (2.3)	1.1 (0.7)	0.7 (0.3)	5.8 (2.0)	13.4 (4.1)	2.3
	Cyst fluid	1.0 (0)	8.0 (0)	0.011 (0.002)	0.031 (0.004)	0.069 (0.011)	0.50 (0.03)	7.3
Tissues	Liver	1.1 (0.9)	3.1 (4.3)	3.1 (2.7)	4.1 (3.8)	10.9 (11.1)	19.4 (3.6)	1.8
	Lung	7.3 (7.5)	4.0 (3.4)	12.1 (12.0)	50.2 (14.2)	49.6 (47.6)	459.7 (133.1)	9.3
	Spleen	2.1 (1.8)	11.3 (8.1)	4.7 (2.1)	10.6 (1.4)	37.5 (28.0)	135.3 (49.5)	3.6
	Kidney	1.2 (0.8)	9.2 (6.1)	7.3 (2.1)	7.6 (2.0)	94.1 (24.4)	137.8 (38.9)	1.5
	Intestine	3.0 (1.7)	1.1 (0.9)	44.0 (39.2)	7.8 (1.8)	256.2 (233.9)	28.2 (13.6)	0.1
	Heart	13.4 (12.1)	3.5 (4.0)	2.4 (1.4)	3.3 (0.6)	28.2 (4.0)	43.9 (4.1)	1.6
	brain	8.7 (7.0)	6.0 (3.5)	0.2 (0.3)	0.5 (0.1)	0.7 (0.3)	3.8 (0.06)	5.4

a: T_max_ was time to the drug peak concentration.

b: C_max_ was the peak concentration of the drug.

c: AUC_0-t_ was the area under the drug concentration-time curve.

d: F (relative bioaviability) = AUC_0-t_ of MBZ-1% tragacanth/AUC_0-t_ of MBZ-OS.

### Influence of MBZ-OS on the liver of mice

#### Histopathological changes of the liver

**Figure 2:**
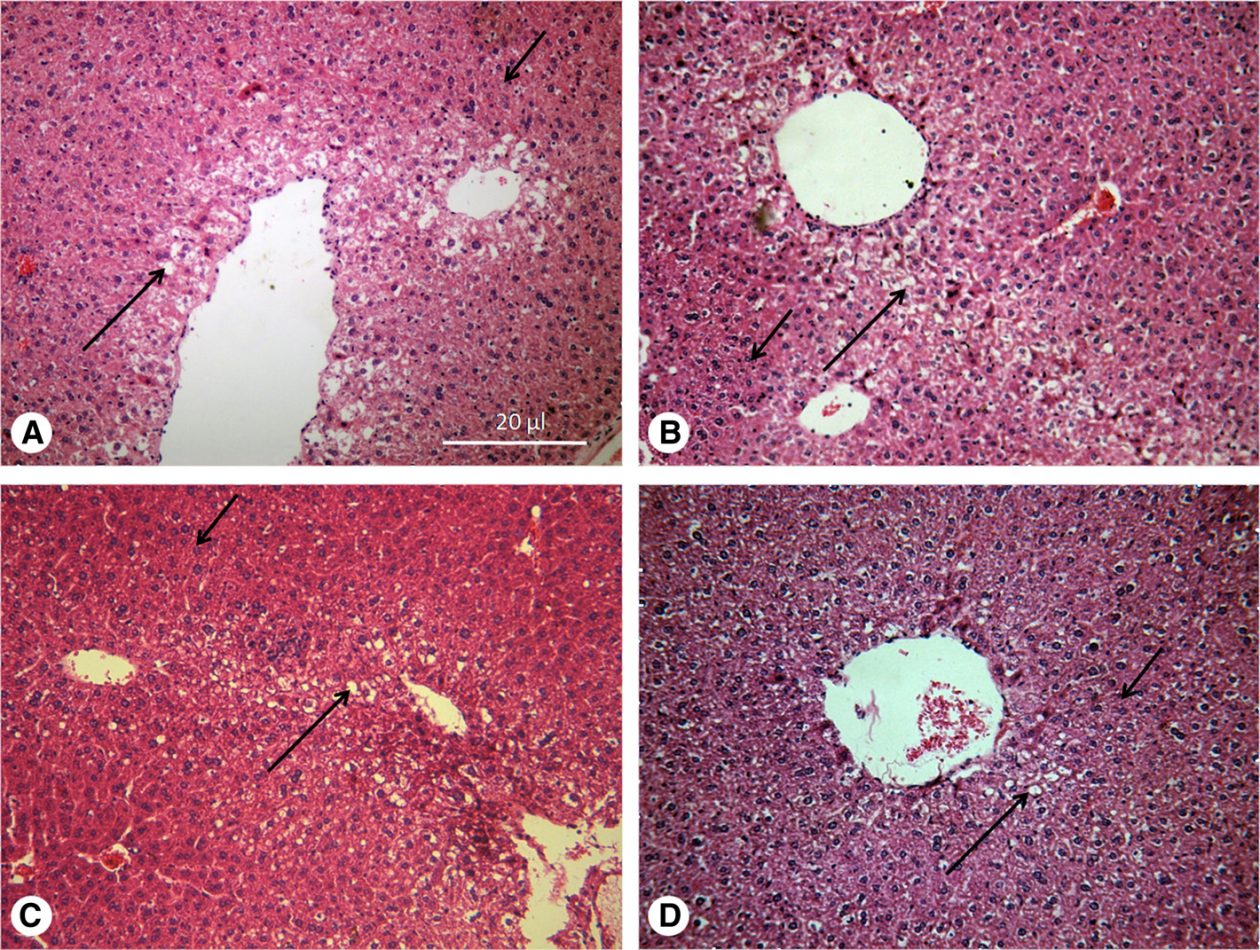
**Histopathological changes of liver.** H + E, fatty infiltration (*large arrow*), hepatocyte swelling (*small arrow*) **A**. Normal mice; **B**. *E. granulosus* infected mice without treatment; **C**. *E. granulosus* infected mice treated with MBZ-1% tragacanth; **D**. *E. granulosus* infected mice treated with MBZ-OS.

After the treatment with MBZ-OS and MBZ-1% tragacanth at 25 mg/kg, histopathological changes in the liver of the normal (uninfected), untreated and treated mice were observed simultaneously (Figure 2, Table 3). Compared with uninfected groups, there was no significant difference from other groups in histopathological changes (*P >* 0.05, Table 3). In uninfected and untreated control groups, the hepatocytes and other cells of the livers were normal and systematically arranged but showed light fatty infiltration. However, fatty infiltration had been seen in all groups without significant difference. The light swelling of hepatocytes was only found in the two treated groups (Figure 2).

**Table 3:** **Histopathological changes in the livers of the mice from the uninfected control, untreated control and treated groups MBZ-1% tragacanth and MBZ-OS (10 mice per group) were observed by microscopy (** ***n*** = **5)**

**Histopathologcial Changes**	**Uninfected control group**	**Untreated control group**	**MBZ-1% tragacanth**	**MBZ-OS**
Congestion	0.0 (0.0)	0.0 (0.0)	0.0 (0.0)	0.0 (0.0)
Fatty infiltration	0.4 (0.3)	0.8 (0.5)*	0.6 (0.6)	0.6 (0.6)
Hepatocyte swelling	0.0 (0.0)	0.0 (0.0)	0.5 (0.7)	0.4 (0.6)
Inflammatory cell infiltration	0.0 (0.0)	0.0 (0.0)	0.0 (0.0)	0.0 (0.0)

*P < 0.05 vs. uninfected group.

#### Liver function test

As seen in Table 4, the liver function test results suggested that the IBIL values of the untreated control group and two treated groups were all significantly increased compared with the uninfected group. In addition, the AST and ALT values of the untreated control group were also significantly higher than that of the uninfected group. However, after the treatment with MBZ-OS or MBZ-1% tragacanth, the values of these two parameters (AST and ALT) were decreased and reached to the level of the uninfected control. It was also found that the GLB value of the MBZ-OS was 28.3 ± 2.4 g/L, significantly higher than that of the other groups.

**Table 4:** **Outcomes of liver function tests for mice from the uninfected control, untreated control and treated groups MBZ-1% tragacanth and MBZ-OS (10 mice per group)**

**Parameters**	**Definitions**	**Units**	**Uninfected control**	**Untreated control**	**MBZ-1% tracangth**	**MBZ-OS**
T-BIL	Total bilirubin	umol/L	2.3 (0.8)	2.8 (0.4)	3.2* (0.8)	2.9 (0.5)
D-BIL	Direct bilirubin	umol/L	0.5 (0.3)	0.3 (0.1)	0.4 (0.2)	0.3 (0.1)
I-BIL	Indirect bilirubin	umol/L	1.8** (0.7)	2.6* (0.4)	2.8* (0.7)	2.6* (0.5)
TP	Total protein	g/L	55.2 (1.9)	57.7 (2.9)	58.2 (3.8)	63.1** (3.2)
ALB	Albumin	g/L	31.6 (1.7)	32.2 (1.6)	32.7 (2.0)	34.8 (2.0)
GLB	Globulin	g/L	23.6 (0.8)	25.6 (3.3)	25.5 (3.0)	28.3** (2.4)
A/G			1.3 (0.1)	1.3 (0.2)	1.3 (0.2)	1.2 (0.4)
AST	Aspartate transaminase	U/L	122.8** (32.1)	173.7* (107.0)	113.9** (16.9)	100.8** (14.7)
ALT	Alanine transaminase	U/L	45.1** (21.8)	81.0* (48.8)	35.7 ** (17.7)	27.6 ** (4.5)
AST/ALT			3.0 (0.8)	2.3 (0.7)	3.6 ** (1.2)	3.7** (0.7)
ALP	Alkaline phosphatase	U/L	86.2 (29.6)	67.4 (14.0)	74.5 (28.8)	80.7 (19.1)

*P < 0.05 vs. uninfected group.

**P < 0.05 vs. untreated control group.

## Discussion

In our previous studies, it was found that, due to the increase of the drug solubility, suspending the insoluble MBZ with oily solvents significantly improved the treatment efficacy against secondary *E. granulosus* cysts than MBZ-1% tracangth [18]. Then the optimal MBZ oily suspension was chosen by comparing the pharmacokinetic parameters, especially the values of C_max_ and AUC_0-t_ (unpublished). At a dose of 25 mg/kg and 12.5 mg/kg, the cyst weight reductions of the MBZ-OS groups were higher than 80%, while MBZ-1% tragacanth was lower than 30%. In some studies, 25 mg/kg was considered as the minimum effective dose for MBZ in the treatment of mice infected with *E. granulosus* cysts [26,34]. However, the lower dosage used in this study made it possible to reduce the oral dose of MBZ-OS in the future clinical trials. At present, the current solid tablet of MBZ is sub-optimal in efficacy, resulting in the need for higher doses and prolonged duration of treatment (40 mg/kg-50 mg/kg per day in three divided doses for at least 3–6 months) [9]. With such long term chemotherapy, the patients hardly continue the treatment as they are more likely to comply with ABZ because it is more cost-effective and involves fewer tablets. However, in view of the fact that the effect of MBZ against *E. granulosus* metacestodes *in vitro* and in experimental therapy proceeded in mice were both better than ABZ [11,12,26,27], it could be concluded that once the absorption problem of MBZ is solved , MBZ would be more widely used with improved clinical cure rate.

The results from the pharmacokinetic analysis of MBZ in plasma, parasite and tissues of mice proved that the improved treatment efficacy was resulted from the higher drug concentration in plasma and parasite. Because of the special structure of the hydatid cysts, MBZ could penetrate these cysts by passive diffusion only [11]. Therefore, the drug concentrations in cyst fluid and cyst wall were determined by that in the plasma. And in MBZ-OS groups, both of the drug levels in plasma and parasite increased. That was, the AUC_0-t_ values of plasma, cyst fluid and cyst wall were 7.1, 7.3 and 2.3 times of that in MBZ-1% tracangth group, while the C_max_ values were 5.9, 2.8 and 0.6 times respectively. It was also found that the MBZ concentration in cyst fluid was obviously lower than in plasma after a single administration of MBZ-OS. However, drug concentration in cyst fluid would be closed to that in plasma by continued chemotherapy, so the long time regime is necessary [35]. In addition, the increased AUC_0-t_ values of cyst fluid and cyst wall in MBZ-OS group were partially due to the prolonged T_max_.

In this study, the pharmacokinetic analysis was proceeded with *E. granulosus* –infected mice. And the calculated pharmacokinetic parameters were numerically different from that of the uninfected mice from our former studies [18]. In details, the C_max_ and AUC_0-t_ values of infected mice were lower than that of uninfected mice with prolonged T_max_. Similarly, Witassek *et al.* reported the MBZ plasma concentration of the *E. multilocularis-*infected jirds was lower than the non-infected ones [36]. It seems that the *E. granulosus* infection might influence the MBZ metabolism in mice. However, the mechanism would be clarified by further study on this topic. In our previous studies, the T_max_ values of MBZ oily suspensions changed a little compared with the MBZ-1% tracangth [18]. These results suggested that the absorption rate which is relevant to the value of T_max_, still limit the absorption of MBZ into the plasma.

After oral administration of MBZ-OS, the drug concentrations in lung, spleen kidney, heart and brain were higher than that of the MBZ-1% tragacanth. However, contributed by the accumulated indissolvable parent drug, the C_max_ value of MBZ-1% tragacanth was nearly 6 times of the new formulation in the intestine. On the other hand, MBZ-OS promoted more MBZ into the systemic circulation. It was found that the C_max_ and AUC_0-t_ values of MBZ-OS in liver and kidney were not changed as much as in plasma. As the crucial metabolism and elimination organs, liver and kidney might clear away MBZ by their corresponding functions. However, in lung, spleen and brain, C_max_ and AUC_0-t_ of MBZ-OS were significantly increased than MBZ-1% tragacanth. Given that the high drug concentration in tissues may bring some safety concern, MBZ-OS was orally given to 10 mice (5 female mice and 5 male mice) at 5 g/kg. After 7 days’ observation, no mice died. Although no acute toxicity of MBZ-OS had been seen in this experiment, a long-term toxicity test is still necessary in the following study.

In the treatment of hydatid diseases, the benzimidazoles were considered safe with occasional side effects [37-39]. In China, albendazole-emulsion is the dominant clinical formulation for patients with echinococcosis. However, patients often feel uncomfortable in the liver after taking albendazole-emulsion on a daily basis. This may be due to the drug side effects or oils added in formulation. Considering that oils were also added in MBZ-OS, then the influence of this MBZ new formulation on the liver of mice was also observed in this study. At 25 mg/kg, the hepatocytes of mice showed light fatty infiltration in all the experimental groups and the light swelling was found in both of the treated groups. So it was suggested that the long term taking of MBZ affected the hepatocytes. But comparing with the uninfected group, the changes were not significant. Hence, it could be preliminary concluded that the liver of mice were not seriously injured by taking MBZ and MBZ-OS than the raw materials. In order to obtain more information about liver toxicity, the liver function test was carried out by collecting the serum of the uninfected, the untreated control, MBZ-1% tragacanth and MBZ-OS group. Unlike the results from the histopathological changes, some parameters showed significantly difference between uninfected group and the other groups. In details, the values I-BIL, AST and ALT of infected mice without treatment were statistically higher than that of uninfected mice. And the orally administration with MBZ did not change the increased I-BIL values but reduced the values of AST and ALT. In addition, the GLB values of the MBZ-OS were significantly increased after taking MBZ-OS. Due to the lack of the normal ranges for KM mice, the real injury of the liver could not be concluded from the liver function test. However, if the significant differences of these parameters between uninfected mice and infected could make sense, I-BIL, AST and ALT values might be used as the indicators for diagnosing the hydatid diseases. But this assumption needed verification with abundant clinical cases.

## Conclusion

In the present study, a promising new formulation of MBZ for treatment of hydatid diseases was developed. This MBZ-OS improved the treatment efficacy in *E. granulosus*-infected mice and no serious injury on the liver was observed. However, based on the increased MBZ concentration, the lung and spleen toxicity should be paid more attention in a future study. For the clinical trials in healthy volunteers and patients, further researches will be required to apply under the support of government and pharmaceutical companies.

## Additional file

Additional file 1:
**Supporting information,** **Figure S1-S2 and Table S1-S3.** An Alternative Mebendazole Formulation for Cystic Echinococcosis: the Treatment Efficacy, the Pharmacokinetics and the Safety on mice.

## Abbreviations

MBZ-OS: Mebendazole oily suspension
MBZ-1% tragacant: Mebendazole suspended in 1% tragacanth
CE: Cystic echinococcosis
PAIR: Puncture, aspiration, injection, re-aspiration
ABZ: Albendazole
MBZ: Mebendazole
AUC_0-t_: The area under the drug concentration-time curve
C_max_: The peak concentration of the drug
T_max_: Time to the drug peak concentration
F: Relative bioavailability
TP: Total protein
ALB: Albumin
GLB: Globulin
TBIL: Total bilirubin
DBIL: Direct bilirubin
IBIL: Indirect bilirubin
ALP: Alkaline phosphatase
ALT: Alanine transaminase
AST: Aspartate transaminase
H + E: Hematoxylin and eosin
