# Supplementary figures and images for: An alternative mebendazole formulation for cystic echinococcosis: the treatment efficacy, pharmacokinetics and safety in mice
Source: Parasit Vectors. 2014 Dec 10;7:589. doi: 10.1186/s13071-014-0589-0 (PMC4273425; doi:10.1186/s13071-014-0589-0)

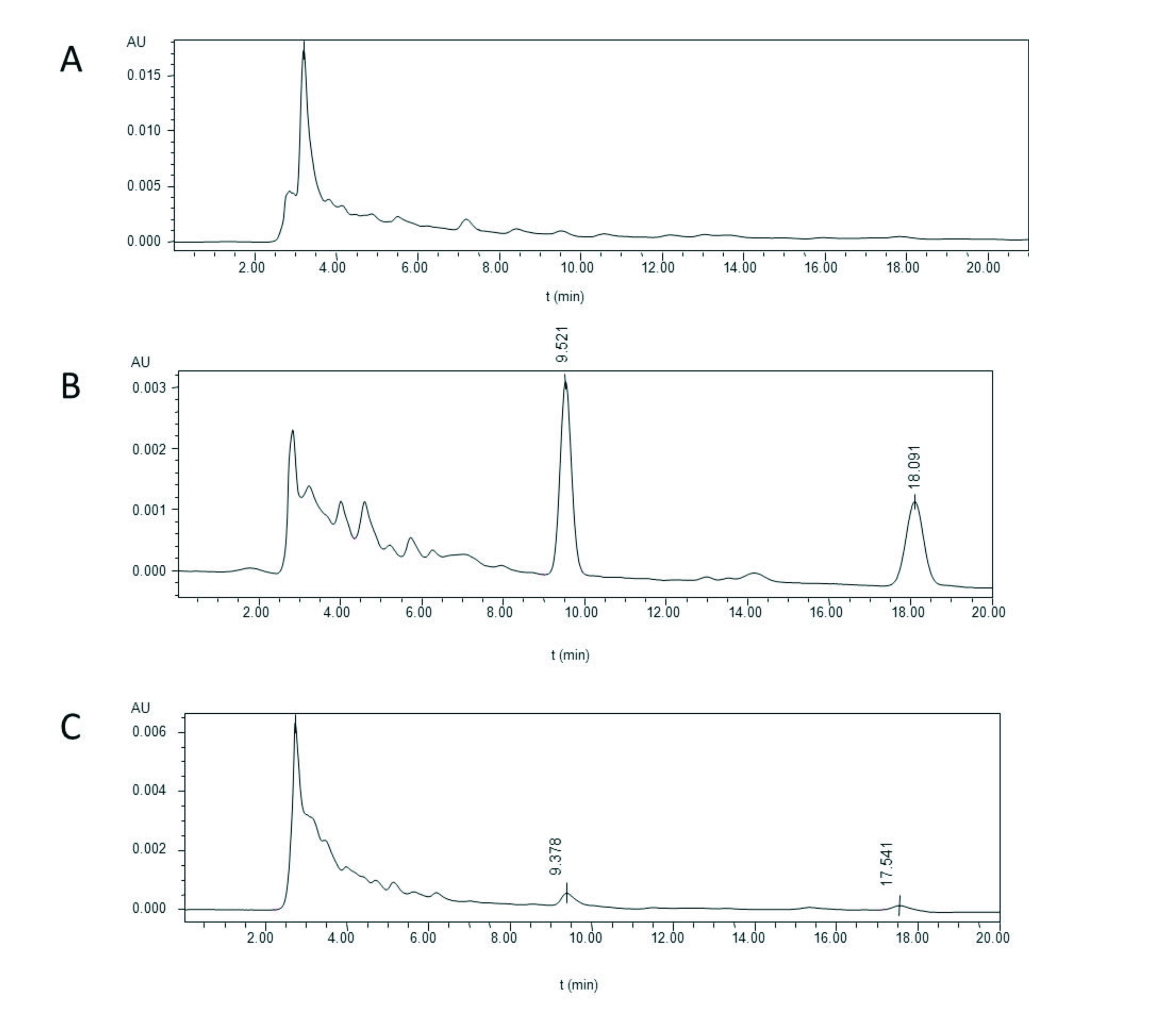

Supplement: Additional file 1: — Supporting information, Figure S1-S2 and Table S1-S3. An Alternative Mebendazole Formulation for Cystic Echinococcosis: the Treatment Efficacy, the Pharmacokinetics and the Safety on mice. [file 13071_2014_589_MOESM1_ESM.zip › S-Figure2.tif]

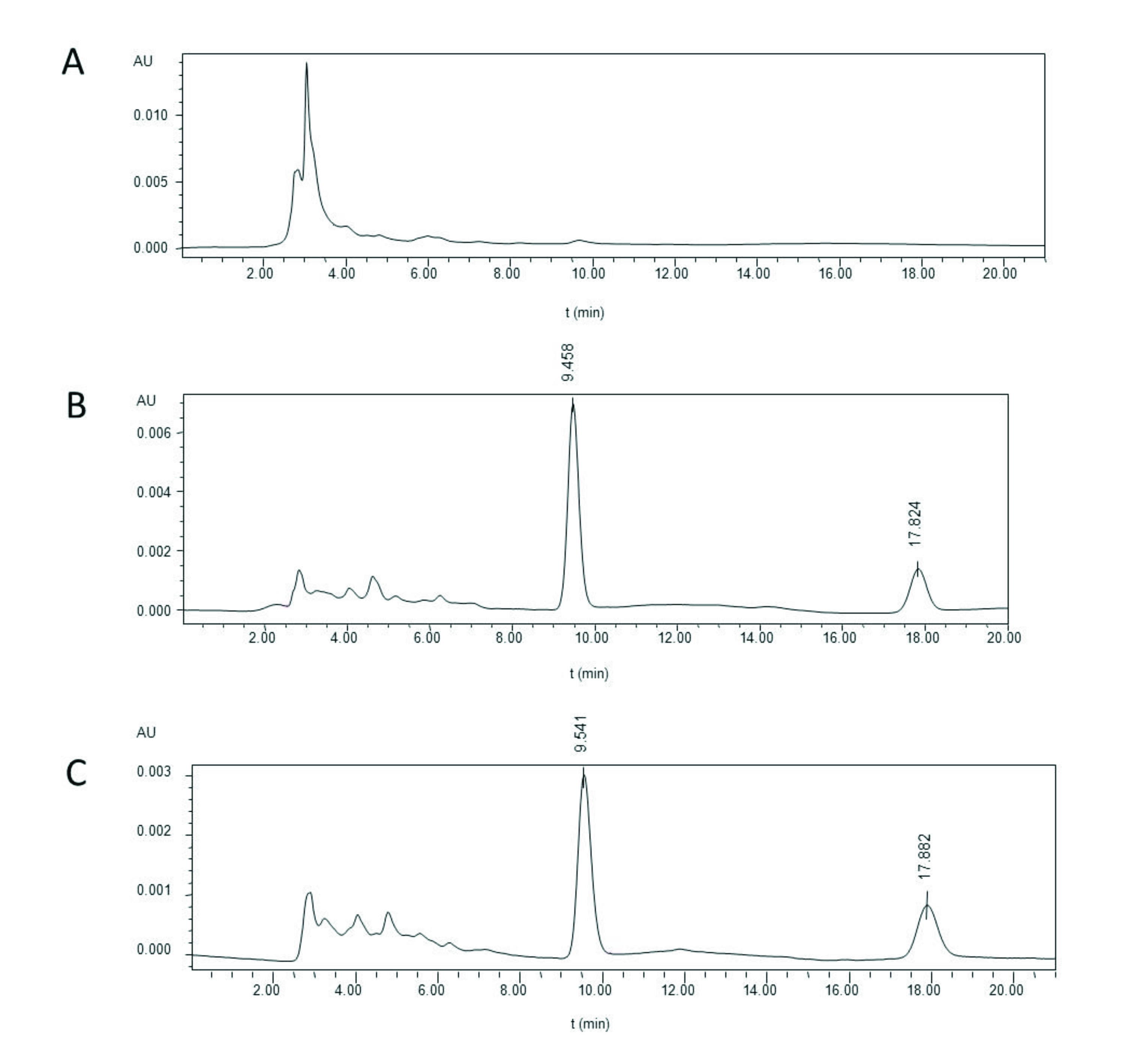

Supplement: Additional file 1: — Supporting information, Figure S1-S2 and Table S1-S3. An Alternative Mebendazole Formulation for Cystic Echinococcosis: the Treatment Efficacy, the Pharmacokinetics and the Safety on mice. [file 13071_2014_589_MOESM1_ESM.zip › S-Figure1.tif]
