# Supplementary material for: An alternative mebendazole formulation for cystic echinococcosis: the treatment efficacy, pharmacokinetics and safety in mice
Source: Parasit Vectors. 2014 Dec 10;7:589. doi: 10.1186/s13071-014-0589-0 (PMC4273425; doi:10.1186/s13071-014-0589-0)
Supplement: Additional file 1: — Supporting information, Figure S1-S2 and Table S1-S3. An Alternative Mebendazole Formulation for Cystic Echinococcosis: the Treatment Efficacy, the Pharmacokinetics and the Safety on mice. [file 13071_2014_589_MOESM1_ESM.zip › Supporting information.doc]

**Supporting information**

**An Alternative Mebendazole Formulation for Cystic Echinococcosis: the Treatment Efficacy, the Pharmacokinetics and the Safety on mice**

Cong-Shan Liu^1^, Hao-Bing Zhang^1*^, Wen Lei^2^, Chao-Wei Zhang^1^, Bin Jiang^1^, Qi Zheng^1^, Jian-Hai Yin ^1^, Xiu-Min Han^2^

**HPLC Determination**

Chromatographic equipment and conditions

The HPLC system (Waters, USA) of instrument consisted of a 1525 Binary HPLC Pumps, a 717 plus auto sampler, a 2457 Dual λ Absorbance Detector and a C_18_ column (250×4.6 mm, 5 μm) (Beckman Coulter, USA). The mobile phase contained 350 ml of 0.05 M ammonium sulfate and 650 ml of methanol at a flow rate of 1ml/min. The mebendazole (MBZ) was detected by a UV detector at 289 nm.

Validation of assay method

*Specificity*

The selectivity of the method was investigated by comparing the UV chromatograms of blank plasma/homogenate of the heart (as a representative sample), plasma and heart samples spiked with MBZ, and plasma and heart samples at 1 h after orally administration of MBZ oily suspension (MBZ-OS) in Figures 1 and 2, respectively. The retention time of MBZ was at about 9.5 minutes and for internal standard (albendazole, ABZ) was 11.5 minutes. No significant peak was observed at or near the retention time of MBZ and ABZ, suggesting that the extraction procedure was capable of obtaining highly purified samples and ensured a high selectivity of the HPLC method.

*Linearity*

The linearity of the calibration curve was determined by plotting the peak area ratio of MBZ to ABZ (y) versus the nominal concentration (x) of MBZ. The calibration curves of MBZ were linear (0.1~10 μg/ml) in plasma, cyst fluid and tissues. The calibration lines of MBZ were calculated by using EXCEL 2000 and shown in Table 1.

*Precision and accuracy*

To determine the within-day accuracy and precision of the method, plasma, cyst fluid and tissues at three concentrations (0.1, 1.0 and 10 μg/ml) were individually analyzed five times in the same day. To determine the between-day accuracy, samples of plasma, cyst fluid and tissues at 0.1, 1.0 and 10 μg/ml were determined by HPLC at five different days.

The intraday and interday precision at three concentrations in plasma, cyst fluid and tissues were listed in Tables 2. Our results indicate that the accuracy of the current assay is suitable for further experiments.

*Extraction recoveries*

The extraction recoveries of MBZ were determined at low (0.1 μg/ml), medium (1 μg/ml), and high (10 μg/ml) concentrations. Then samples of plasma, cyst fluid and tissues were prepared and analyzed as described in the main document. And the same concentration standard solution was injected directly to the HPLC system as the reference material. The extraction recoveries were obtained by comparing the areas ratios of those two samples to the internal standard. The experiments were repeated three times at 0.1, 10, and 100 μg/ml of MBZ, respectively. The summaries of the mean recoveries are listed in Tables 3. The results show that the extraction recoveries of MBZ were different among plasma, cyst fluid and tissues, ranging from 41.8 % to 91.4 %.

**Supporting Infromation Legends:**

**HPLC Determination**

**Figure Legends：**

**Figure 1. Chromatograms of MBZ and ABZ (internal standard) in plasma:** (A) blank plasma; (B) blank plasma with MBZ at 1 μg/ml; and (C) plasma sample after orally administration of MBZ-OS.

**Figure 2. Chromatograms of MBZ and ABZ (internal standard) in heart:** (A) blank homogenate of heart; (B) blank homogenate of heart with MBZ at 1 μg/ml; and (C) blank homogenate of heart after orally administration of MBZ-OS.

**Table Legends:**

Table 1. Standard curve of MBZ in plasma and different tissues

| Plasma, Parasite  and Tissues | | Standard Curve | R^2^ |
| --- | --- | --- | --- |
| Plasma |  | y=4.560x+3.130 | 0.999 |
| Parasite | Cyst wall | y=7.557x-0.192 | 0.999 |
|  | Cyst fluid | y=4.327x+0.110 | 0.999 |
| Tissues | Heart | y=15.08x+0.710 | 0.998 |
|  | Liver | y=1.650x+0.007 | 0.998 |
|  | Spleen | y=4.266x+0.855 | 0.937 |
|  | Lung | y=0.926x+0.751 | 0.999 |
|  | Kidney | y=1.840x+0.879 | 0.990 |
|  | Intestine | y=2.991x+0.455 | 0.995 |
|  | Brain | y=2.127x+0.943 | 0.990 |

Table 2. Intraday and interday precision of the method for determination of MBZ in mice plasma, parasite and tissues samples

| Plasma, Parasite  and Tissues | | Add concentration  (μg/ml) | Precision^a^ (%) | |
| --- | --- | --- | --- | --- |
|  |  |  | Intraday (*n*=5) | Interday (*n*=5) |
| plasma |  | 10 | 1.22 | 2.42 |
|  |  | 1 | 0.70 | 1.85 |
|  |  | 0.1 | 1.13 | 2.24 |
| Parasite | Cyst wall | 10 | 1.09 | 1.25 |
|  |  | 1 | 2.69 | 2.01 |
|  |  | 0.1 | 1.12 | 1.54 |
|  | Cyst fluid | 10 | 0.42 | 1.86 |
|  |  | 1 | 0.71 | 1.54 |
|  |  | 0.1 | 0.99 | 1.25 |
| Tissues | Heart | 10 | 0.97 | 1.22 |
|  |  | 1 | 1.77 | 2.40 |
|  |  | 0.1 | 1.66 | 2.12 |
|  | Liver | 10 | 0.97 | 1.15 |
|  |  | 1 | 2.39 | 2.85 |
|  |  | 0.1 | 3.80 | 2.32 |
|  | Spleen | 10 | 1.34 | 1.04 |
|  |  | 1 | 3.22 | 3.35 |
|  |  | 0.1 | 3.29 | 3.54 |
|  | Lung | 10 | 1.10 | 2.12 |
|  |  | 1 | 3.08 | 2.89 |
|  |  | 0.1 | 3.59 | 3.55 |
|  | Kidney | 10 | 0.61 | 1.02 |
|  |  | 1 | 1.85 | 1.52 |
|  |  | 0.1 | 2.57 | 2.37 |
|  | Intestine | 10 | 3.23 | 2.82 |
|  |  | 1 | 2.70 | 1.98 |
|  |  | 0.1 | 0.37 | 1.12 |
|  | Brain | 10 | 0.63 | 1.05 |
|  |  | 1 | 1.08 | 2.33 |
|  |  | 0.1 | 1.25 | 2.24 |

^a^Expressed as relative standard deviation (SD).

Table 3. Extraction recoveries data of MBZ in mice plasma, parasite and tissues samples (*n*=3)

| Plasma, Parasite  and Tissues | | Add concentration  (μg/ml) | Extraction recoveries^a^  (%) | RSD^b^  (%) |
| --- | --- | --- | --- | --- |
| plasma |  | 10 | 85.4 | 2.3 |
|  |  | 1 | 83.2 | 1.5 |
|  |  | 0.1 | 83.7 | 2.1 |
| Parasite | Cyst fluid | 10 | 94.4 | 3.2 |
|  |  | 1 | 90.1 | 1.7 |
|  |  | 0.1 | 91.1 | 1.9 |
|  | Cyst wall | 10 | 88.2 | 3.3 |
|  |  | 1 | 85.7 | 1.9 |
|  |  | 0.1 | 85.9 | 1.2 |
|  | Heart | 10 | 70.9 | 9.1 |
| Tissues |  | 1 | 76.0 | 2.1 |
|  |  | 0.1 | 71.2 | 3.0 |
|  | Liver | 10 | 62.5 | 0.7 |
|  |  | 1 | 65.8 | 2.7 |
|  |  | 0.1 | 63.1 | 1.5 |
|  | Spleen | 10 | 57.8 | 0.5 |
|  |  | 1 | 53.8 | 3.7 |
|  |  | 0.1 | 50.4 | 2.8 |
|  | Lung | 10 | 87.9 | 9.5 |
|  |  | 1 | 83.2 | 2.1 |
|  |  | 0.1 | 85.6 | 0.4 |
|  | Kidney | 10 | 46.6 | 6.6 |
|  |  | 1 | 41.8 | 4.5 |
|  |  | 0.1 | 45.8 | 2.2 |
|  | Intestine | 10 | 58.4 | 2.3 |
|  |  | 1 | 51.6 | 1.8 |
|  |  | 0.1 | 51.7 | 4.4 |
|  | Brain | 10 | 84.5 | 4.0 |
|  |  | 1 | 88.8 | 3.1 |
|  |  | 0.1 | 86.2 | 2.6 |

^a^Expressed as [(mean observed concentrations/nominal concentrations) × 100].

^b^Expressed as relative standard deviation (SD).
